# Root-endophytes improve the ecophysiological performance and production of an agricultural species under drought condition

**DOI:** 10.1093/aobpla/plw062

**Published:** 2016-10-27

**Authors:** Marco A. Molina-Montenegro, Rómulo Oses, Cristian Torres-Díaz, Cristian Atala, Andrés Zurita-Silva, Simón Ruiz-Lara

**Affiliations:** 1 Instituto de Ciencias Biológicas, Universidad de Talca, Avenida Lircay s/n, Talca, Chile; 2 Núcleo Milenio “Centro en Ecología Molecular y Aplicaciones Evolutivas en Agroecosistemas (CEM)”, Avda. Lircay s/n, Talca, Chile; 3 Centro de Estudios Avanzados en Zonas Áridas (CEAZA), Facultad de Ciencias del Mar, Universidad Católica del Norte, Larrondo #1281, Coquimbo, Chile; 4 Research Program “Adaptation of the Agriculture to Climate Change” PIEI A2C2, Universidad de Talca, Talca, Chile; 5 Laboratorio de Genómica & Biodiversidad (LGB), Departamento de Ciencias Naturales, Universidad del Bío-Bío, Chillan, Chile; 6 Laboratorio de Anatomía y Ecología Funcional de Plantas, Instituto de Biología, Facultad de ciencias, Pontificia Universidad Católica de Valparaíso, Campus Curauma, Valparaíso, Chile; 7 Instituto de Investigaciones Agropecuarias (INIA), Centro de Investigación Intihuasi, Colina San Joaquín s/n, La Serena, Chile

**Keywords:** Drought tolerance, lettuce, NHX1 gene expression, symbiosis, water use efficiency

## Abstract

Throughout many regions of the world, climate change has limited the availability of water for irrigating crops. Indeed, current models of climate change predict that arid and semi-arid zones will be places where precipitation will drastically decrease. In this context, plant root-associated fungi appear as a new strategy to improve ecophysiological performance and yield of crops under abiotic stress. Thus, use of fungal endophytes from ecosystems currently subjected to severe drought conditions could improve the ecophysiological performance and quantum yield of crops exposed to drought. In this study, we evaluated how the inoculation of fungal endophytes isolated from Antarctic plants can improve the net photosynthesis, water use efficiency and production of fresh biomass in a lettuce cultivar, grown under different water availability regimes. In addition, we assessed if the presence of biochemical mechanisms and gene expression related with environmental tolerance are improved in presence of fungal endophytes. Overall, those individuals with presence of endophytes showed higher net photosynthesis and maintained higher water use efficiency in drought conditions, which was correlated with greater fresh and dry biomass production as well as greater root system development. In addition, presence of fungal endophytes was correlated with a higher proline concentration, lower peroxidation of lipids and up-/down-regulation of ion homeostasis. Our results suggest that presence of fungal endophytes could minimize the negative effect of drought by improving drought tolerance through biochemical mechanisms and improving nutritional status. Thus, root-endophytes might be a successful biotechnological tool to maintain high levels of ecophysiological performance and productivity in zones under drought.

## Abstract (precis)

Antarctic root-endophytes can improve the physiological tolerance and productivity of lettuce crops by means of several different physiological and molecular mechanisms, and could be a successful strategy to maximize water use efficiency and hence maintain an optimal yield in zones affected by desertification. Thus, application of antarctic root-endophytes to different crops could be a biotechnological tool for food security.

## Introduction

Climatic variations are widely indicated as a major driver for global food security (Fedoroff *et al.* 2010). Considering the current models of climate change, decrease in soil water availability will be a conspicuous constraint for both native plant communities as well as several crops worldwide (IPCC, 2013). Hence, lands under osmotic stress (due to salinity and/or drought) are expected to increase during this century (Fedoroff *et al.* 2010), decreasing food production, especially in those areas where a drastic decrease in precipitation has been predicted, or is already occurring at high rates (Martínez *et al.* 2009). For example, it has been estimated that over 930 million hectares of arable lands are affected by salinity and water stress worldwide (FAO, 2008).

Water shortage already is one of the most common factors of stress that affects and limits agricultural productivity worldwide (Sánchez, 2000; Pessarakli, 2007; Acar *et al.* 2008). Several crops have high water demands and they must be supplemented with additional irrigation. However, water available for irrigation has become limited in many countries as a result of global climate change (Hamdy *et al.* 2003; Flexas *et al.* 2006). Many physiological, and some morphological strategies allowing different crops to reduce the negative effects of water shortage have been documented (Nobel, 2005; Flexas *et al.* 2006). Indeed, plant responses and mechanisms for dealing with water shortage can be divided into two major strategies: stress avoidance and stress tolerance (Claeys and Inze, 2013). In stress avoidance, water uptake is enhanced by accumulation of solutes to lower tissue water potential and by increasing root growth, while water loss is limited by closing stomata, restricting shoot growth and modifying leaf senescence. Tolerance mechanisms are aimed at protecting against cell damage when stress becomes more severe and avoidance mechanisms are insufficient. This last strategy includes detoxiﬁcation of reactive oxygen species (ROS) and the accumulation of solutes such as proline, which has a dual role as both osmolyte and osmoprotectant (Claeys and Inze, 2013). This compound accumulates in response to many abiotic stresses and acts as a regulator of redox balance and signalling molecule. Proline is also considered one of the main osmolytes able to scavenge free radicals, thereby ensuring membrane stabilization and preventing protein denaturation during severe osmotic stress (Szabados and Savouré, 2010; Shabala *et al.* 2012). Since different stresses share common osmotic responses, accumulation of sugars, proline and other ions such as K^+^^ ^allows plants to maintain the cellular turgor pressure necessary for cell expansion under stress conditions (McCubbin *et al.* 2014).

In addition, the presence of root-associated fungi has been reported in several crops as another important strategy to maintain or improve ecophysiological performance and/or yield (Thom *et al.* 2012; Coleman-Derr and Tringe, 2014), but this has been seldom experimentally tested. For example, fungal endophytes have been shown to confer drought-tolerance in many crops by morphological and biochemical mechanisms such as higher water use efficiency and photosynthetic rate (Swarthout *et al.* 2009), osmoprotectants or compatible solutes (Grover et al. 2011), improving the nutritional status and root growth (Malinowski and Belesky, 2000) and driving the expression of genes implied in homeostasis (Estrada *et al.* 2013). In extreme environments, like those found on the Antarctic continent, fungal endophytes have been shown to provide benefits to resident vegetation exposed to harsh conditions like extreme cold, low nutrient availability and water shortage (Upson *et al.* 2009). Thus, Antarctic endophytes associated to roots could be a useful strategy for plants to cope with water stress present in arid environments or under drought conditions (Fardella *et al.* 2014). Although, several studies have shown the positive effect of mycorrhiza on plant physiology and abiotic tolerance (Ruiz-Lozano, 2003*;* Ruiz-Lozano *et al.* 2012; Coleman-Derr and Tringe, 2014), as far as we know, our study is the first to assess the effects of Antarctic root-endophytes on physiological performance, quantum yield and environmental tolerance in a crop.

Lettuce (*Lactuca sativa*) is one of the most sensitive crop to water deficit (Sánchez, 2000; Boroujerdnia et al. 2007; Molina-Montenegro *et al.* 2011). This crop has been shown to be highly dependent on water at all developmental stages, and demands constant watering to maintain high photosynthetic rates and a fresh biomass of high commercial value (Sánchez, 2000; Nissen and San Martín, 2004). Thus, this crop could be suitable to test the effect of Antarctic root-endophytes as a potential biotechnological solution to lessen the negative effects of water shortage on cultivated plants.

In this study, we assessed whether lettuce plants inoculated with Antarctic root-endophytes improve abiotic stress tolerance and ecophysiological performance compared with non-inoculated plants exposed to drought conditions. Specifically, we tested if inoculation with root-endophytes isolated from Antarctic plants confer morphological and biochemical mechanisms that enhances water use efficiency, photosynthesis, as well as shoot and root biomass in a lettuce crop. Finally, we assessed if expression of a gene implicated in the regulation of ion homeostasis (NHX1), protection of cellular membranes and proline concentration is enhanced in plants of lettuce when inoculated with the Antarctic root-endophytes and exposed to drought.

## Methods

### Isolation and identification of root-endophytes

Twenty-five fresh roots of *Colobanthus quitensis* (Caryophyllaceae) and 25 fresh roots of *Deschampsia antarctica* (Poaceae) were collected with a cluster of soil around the roots (approximately 250 g) growing under natural conditions in King George Island, Antarctica (62°09′S; 50°28′W) during the growing season (November 2014–February 2015). The roots were then cut into pieces and stored in plastic bags at 10 °C before isolation of endophytic fungi. The roots were superficially sterilized by successive immersion in ethanol 70 % (1 min) and 2 % sodium hypochlorite (3 min), followed by washing with sterile distilled water (2 min) (Collado *et al.* 1996). Then, fragments of roots were plated on Petri dishes containing potato dextrose agar (PDA, Difco, USA) plus chloramphenicol at 100 g mL^−1^. The Petri dishes with root fragments were incubated up to 60 days at 18 °C. Roots were observed routinely under a dissecting microscope, and the emerging fungi were transferred onto PDA. Hyphae growing out of the root segments were re-inoculated in new plates with fresh medium. Different hyphae growing in the same root fragment that showed similar colony morphology were clustered. The isolates were maintained by routine sub-culturing with single-spore isolations. Finally, individual colonies formed were stored at 4 °C until their utilization in the field experiments.

For molecular identification of isolated root-endophytes, we amplified the ITS (which includes ITS1, ITS2 and the intercalary 5.8S rRNA gene) and LSU (the 28S nuclear ribosomal large subunit rRNA gene) regions. DNA was extracted from mycelia in active growing using E.Z.N.A. fungal DNA MiniKit (Omega-Biotek). ITS region was amplified using ITS5 (5′-GGAAGTAAAAGTCGTAACAAGG-3′) and ITS4 (5′-TCCTCCGCTTATTGATATGC-3′) as forward and reverse primers (White *et al.* 1990), respectively. LSU region was amplified using LR0R (5′-GTACCCGCTGAACTTAAGC-3′) and LR06 (5′-CGCCAGTTCTGCTTACC-3′) as forward and reverse primers respectively (Vilgalys and Hester, 1990). Each PCR reaction was conducted in a 15 µl volume containing 30–50 ng of DNA, of PCR buffer diluted 10 times from stock solution, containing 2 mM MgCl_2_, 0.1 µM of each dNTP, 0.5 µM of forward and reverse primers, and one unit (1 U) of Taq DNA polymerase. PCR amplifications were carried out with an initial denaturation of 4 min at 94 °C, and then 35 cycles of 30 s at 94 °C, 60 s at 50 °C and 60 s at 72 °C s, followed by a final step of 5 min at 72 °C. After this, the PCR product was purified and both strands sequenced with Macrogen sequence service (Seoul, Korea). After sequencing, the fragments of forward and reverse sequences were edited using Geneious v5.4 software (Drummond *et al.* 2011). The sequence of each isolated endophyte was analyzed with MegaBLAST (Basic Local Alignment Search Tool) (http://blast.ncbi.nlm.nih.gov/Blast.cgi) in order to determine the percentage of maximal identity and total scores with the sequences of that global database. Finally, obtained sequences (ITS1-5.8S-ITS2 and 28S) were assembled and deposited in Gene-Bank database.

### Field experiments

Lettuce seedlings (var. Romaine) were obtained from seeds germinated in growth chambers located at Centre for Advanced Studies in Arid Zones (CEAZA), Coquimbo, Chile (29°S), under controlled environmental conditions. For treatment setup, lettuce seedlings were transplanted into the field when each individual presented at least four true leaves and 2 cm-emerged roots. Seedlings were divided into four treatments: (i) 100 % of the water irrigation that each seedling receives typically in the Coquimbo region (40 ml/day), (ii) 75 % of normal water irrigation (30 ml/day), (iii) 100 % of water plus root-endophytes inoculation and (iv) 75 % of water plus root-endophytes inoculation. Endophytes inoculation was done with a concentrated mix of spores (5000 spores g^−^^1^), from the two most abundant endophytes (1:1) isolated in the laboratories at CEAZA. The inoculation was repeated three times to ensure fungal association, and verification of an effective symbiosis was evidenced by microscopy. Before the beginning of the experiment, two plants per treatments were sacrificed to check microscopically for the presence and/or absence of endophytes by routine staining.

The amount of water that is normally added to reach marketable size in lettuce crops at CEAZA experimental station in the Coquimbo region was considered as 100 % of water used to irrigate each plantlet. The seedlings (*n* = 100 for each treatment) were transplanted to the field and distributed in rows at 0.5 m-distance, and separated from each other by 0.2 m over each row planting. Different treatments were transplanted on independent rows (four rows per each treatment). The experimental site is characterized by clay soils of good drainage, and with low levels of salt and macronutrients (data not shown). Each individual was supplemented with 0.2 g L^−^^1^ of Phostrogen^®^ (Solaris, NPK, 14:10:27) every 30 days. The experiment lasted for 100 days, and the measurements were made simultaneously in all treatments. Environmental conditions were recorded at midday (12:00–14:00 h) during all experimental period (from March to May). Air temperature and relative humidity was recorded with a data logger (HOBO-Pro v2 U-23) and sunlight was registered with a portable photosynthetic active radiation sensor (Li-190 quantum sensor). Air temperature and relative humidity reached mean values of 20.4 °C (± 3.3) and 73 % (± 9), respectively; while sunlight reached an average value of 1045 μmol m^−^^2^s^−^^1^ (± 434).

### Ecophysiological traits

The net photosynthesis rate (A), and transpiration rate (E) were measured on a visually healthy leaf from 25 individuals corresponding to each treatment. Measurements were made on the same individual at 30, 60 and 90 days, by an infrared gas analyzer (IRGA, Infra Red Gas Analyser, CIRAS-2, PP-Systems Haverhill, USA). From gas exchange measurements, we estimated the instantaneous water use efficiency (WUE) for photosynthesis as the ratio between photosynthetic rate and transpiration (A/E). This parameter is used as an indicator of plant water stress in a microsite or condition, because an increase in WUE is usually induced by a decrease in water availability (Lambers *et al.* 1998).

At the end of the experimental period, sampled individuals in each treatment were extracted from the soil without damaging the root system. Subsequently, the roots were washed without removing them from the stem and left to dry in the shade for 1 h. Total fresh biomass of both, shoots and roots of each individual was weighed with a digital electronic balance (Boeco BBL-52; 0.01 gr-precision). Finally, total dry biomass was obtained after whole lettuce individuals were over-dried at 62 °C for 96 h.

### Protective mechanisms

In order to assess whether presence of antarctic root-endophytes regulate some parameters related with the potential mechanisms involved in drought tolerance, we measured cell damage by oxidative degradation of lipids and proline levels. Lipid peroxidation is considered an indicator of cell damage, and was estimated by measuring the concentration of malondialdehyde (MDA) by the thiobarbituric acid (TBA) assay (Egert and Tevini, 2002). Twenty-five lettuce plants from each treatment were analysed at the end of experiment. For each seedling, fresh tissue (0.5 g) was extracted and homogenized with 2 ml of TCA (1%) and centrifuged at 10 000 g for 5 min. 250 ml of the supernatant were mixed with 1 ml of TBA (0.5%) in TCA (20%). Mixtures were incubated in boiling water for 30 min, and then cooled to room temperature. Absorbance was determined at 532 nm and non-specific absorbance at 600 nm (Hodges *et al.* 1999). The MDA content was determined by its molar extinction coefficient of 155 mM^−^^1^cm^−^^1^.

Proline concentration in fresh tissues was determined in lettuce plants from all treatments following Bate’s method (Bates *et al.* 1973) with slight modifications. Leaf tissue (100 mg) was frozen and ground in 1.2 ml of 3 % sulphosalicylic acid and the homogenate was centrifuged at 16 000 g at room temperature for 20 min. An aliquot of the supernatant (1 ml) was added to 2 ml ninhydrin reagent [2.5 % ninhydrin in glacial acetic acid–distilled water–85 % orthophosphoricacid (6:3:1)]. The reaction mixtures were kept in a water bath at 90 °C for 1 h to develop the colour. Test tubes were then cooled in an ice-bath, and 2 ml toluene was added to separate the chromophore. Absorbance of the toluene phase was read in a spectrophotometer at 525 nm, and proline concentration was calculated by comparing sample absorbancies with a standard proline curve.

### Quantitative real-time PCR (qRT-PCR) analysis

Total RNA was extracted from shoots of 30 days old seedlings (*n* = 25 ind. per treatment) according to Chang *et al.* (1993). RNA yield and purity were checked by means of UV absorption spectra, whereas RNA integrity was determined by electrophoresis on agarose gel. DNA was removed using TURBO DNA-free (Applied Biosystems, California, USA) from aliquots of total RNA. The first-strand cDNA was synthesized according to previous methods (Ruiz-Carrasco *et al.* 2011). The reaction of quantitative PCR (qPCR) was performed in a final volume of 20 μl containing the cDNA, 5 pmol of each primer and 12.5 µl of the Fast SYBR Green PCR master mix (Applied Biosystems) according to the manufacturer’s instructions. The Elongation Factor 1a (*EF1a*) housekeeping gene was used as reference gene to normalize, and estimate up- or down-regulation of the target genes for all qPCR analyses: 5′-GTACGCATGGGTGCTTGACAAACTC-3′ (forward); 5′-ATCAGCCTGGGAGGTACCAGTAAT-3′ (reverse). *NHX1* sequences were used to amplify *LsNHX1* amplicons that were 200-bp long: 5′-GCACTTCTGTTGCTGTGAGTTCCA-3′ (forward); 5′-TGTGCCCTGACCTCGTAAACTGAT-3′ (reverse).

PCRs were carried out with Step One Plus 7500 Fast (Applied Biosystems) for an initial cycle of 30 min at 45 °C and 2 min at 95 °C, and then 40 cycles as follows: 95 °C for 30 s, 60 °C for 30 s, 72 °C for 2 min and finally one cycle at 72 °C for 10 min. Cycle threshold (Ct) values were obtained and analyzed with the 2^−^^ΔΔC^_T_ method (Livak and Schmittgen, 2001). The relative expression ratio (log_2_) between each target gene and the reference gene, and fold changes (FC) between drought-treated samples vs. corresponding controls were calculated from the qRT-PCR efficiencies and the crossing point deviation using the mathematical model proposed by Pfaffl (2001).

### Nutrient content

Shoot tissue nutrients were determined at the end of experiment on seven individuals from each treatment, and expressed as percentage on the dry weight basis. All analyse were conducted in the Laboratory of Nutrient Analysis at Universidad de Concepción, Chillán. Shoot nutrient concentrations were determined after dry-ashing (except for nitrogen). NO_3_ and NH_4_ were determinated after KCl extraction; P by Bray-1 method; K, Ca, and Mg after ammonium acetate extraction. N was determined via combustion analysis (CNS-2000 Macro Analyzer, Leco Inc., MI, USA). P, K, Ca and Mg were measured by ICP-OES (Perkin Elmer Optima 3000DV, Wellesley, MA, USA).

### Statistical analysis

The effect of root-fungal endophytes on the ecophysiological parameters (net photosynthesis, and water use efficiency) in lettuce plants subject to different watering treatments was analyzed by a one-way repeated-measures ANOVA. We considered the ecophysiological parameters as response variables, different treatments (endophytes and watering) as independent variable and time as the repeated variable. Differences in fresh and dry biomass, peroxidation of lipids, proline accumulation and gene expression were compared by a one-way ANOVA, with endophytes and watering treatment as the independent variables. Similarly, shoot nutrient concentrations were compared by a one-way ANOVA, with endophytes and watering treatment as the independent variables. Differences between treatments were evaluated using an *a posteriori* Tukey test. Normality and variance homogeneity were assessed using the Shapiro–Wilks test and Bartlett test, respectively (Sokal and Rohlf, 1995).

## Results

### Root-endophytes identification

Fourteen different fungal endophytes were isolated from root samples obtained from *Colobanthus quitensis* and *Deschampsia antarctica*. The two most abundant fungal morphotypes (one of each plant species) corresponded to two isolates of ‘Antarctic Fungal Endophytes—AFE’ (AFE001 and AFE002), which represented more than 75 and 88 % of the total fungal morphotypes recorded in *C. quitensis* (AFE001) and *D. antarctica* (AFE002), respectively. The identities of these two strains (AFE001 and AFE002) were confirmed by using MegaBLAST. AFE001 isolate (Genebank code: KJ881371) was identified as *Penicillium chrysogenum* as it showed 100 % of similitude to *P. chrysogenum* accessions both in ITS (e.g. KF5784432.1; total score = 627) and LSU (e.g. KF417576.1; total score = 1705) regions. Similarly, AFE002 isolate (Genebank code: KJ881370) was identified as *Penicillium brevicompactum*, showing 100 % similarity with *P. brevicompactum* accessions both in ITS (e.g. KF156318.1; total score = 670) and LSU (e.g. JN938947.1; total score = 1579) regions.

### Ecophysiological traits

**Figure 1:**
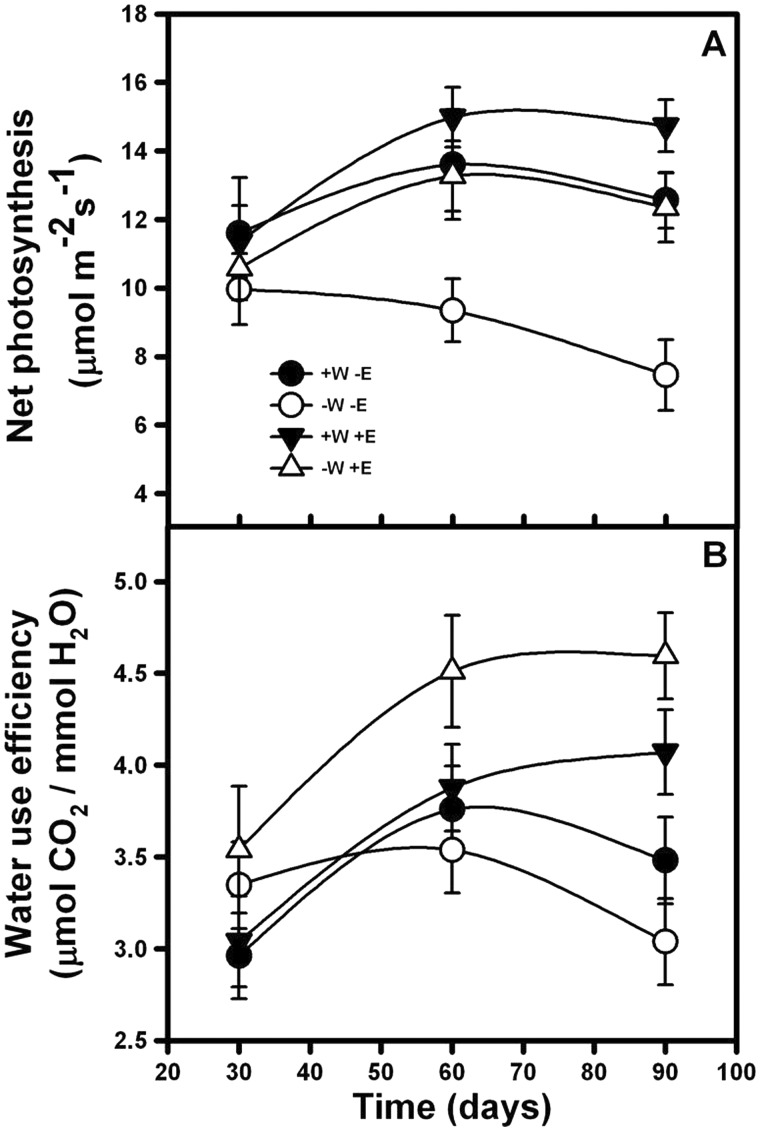
Net photosynthesis (A) and water use efficiency (B) of lettuce plants in time (30, 60 and 90 days). Individuals were subjected to 40 and 30 ml/day of tap water (+W and −W), and with or without presence of root-endophytes (+E and −E) isolated from Antarctic plants. Mean values are shown (± 1 SD).

Net photosynthetic rate (*A*_max_) was affected by root-endophyte presence and watering regime (F_99, 3 _=_ _177.12; *P* < 0.001). Net photosynthesis was significantly higher in the treatments with the presence of root-endophytes at 100 and 75 % of water availability, in comparison with the individuals without root-endophytes (Fig. 1A, Tukey test *P* < 0.05). Additionally, the interaction (Treatment × Time) was significant (F_198, 6 _=_ _40.56; *P* < 0.001), because with increasing exposure time to treatment, photosynthetic rate decreased only in those plants that received 75 % of irrigation without root-endophytes (Fig. 1A).

Watering and endophyte inoculation affected WUE (ANOVA, F_99, 3 _=_ _67.47, *P* = 0.021). Individuals from the treatment with 75 % irrigation plus root-endophytes showed the greatest WUE, followed by those with 100 % irrigation plus root-endophytes and 75 % irrigation without fungi, and finally by control individuals who received 100 % irrigation and no inoculation (Fig. 1B, Tukey test, *P* > 0.05). The ANOVA showed a significant effect in the Treatment × Time interaction factor (F_198, 6 _=_ _32.52; *P* = 0.041). While individuals from the 100 or 75 % irrigation plus root-endophytes treatment increased their water use efficiency over time, those under treatment without root-endophytes maintained or even decreased their WUE (Fig. 1B).

We found significant differences in total fresh biomass between treatments (F_99, 3 _=_ _104.07; *P* < 0.001). Individuals with 100 % irrigation plus root-endophytes had greater fresh biomass production compared to the other treatments (Table 1). Additionally, individuals grown with 75 % irrigation plus root-endophytes showed similar fresh biomass than those grown with 100 % irrigation with no endophytes (Table 1). All treatments showed significantly higher fresh biomass than plants grown with 75 % irrigation and without root-endophytes (Table 1).

**Table 1.** Shoot and root fresh biomass, and total (shoot + root) dry biomass of lettuce plants.

Parameter	+W −E	−W −E	+W +E	−W +E
Shoot fresh biomass (g)	305 (± 15.6) b	204 (± 10.1) c	345 (± 12.5) a	266 (± 22.5) b
Root fresh biomass (g)	15.2 (± 5.1) a	6.1 (± 3.8) b	19.6 (± 7.6) a	24.1 (± 5.7) a
Total dry biomass (g)	36.2 (± 4.6) ab	27.1 (± 4.2) b	40.4 (± 4.1) a	31.1 (± 3.3) b

Individuals were subjected to 40 and 30 ml/day of tap water (+W and −W, respectively), and with or without presence of root-endophytes (+E and −E, respectively) isolated from antarctic plants. Different letters indicate significant differences; tukey test, α < 0.05). mean values are shown (± 1 SD).

### Protective mechanisms

**Figure 2:**
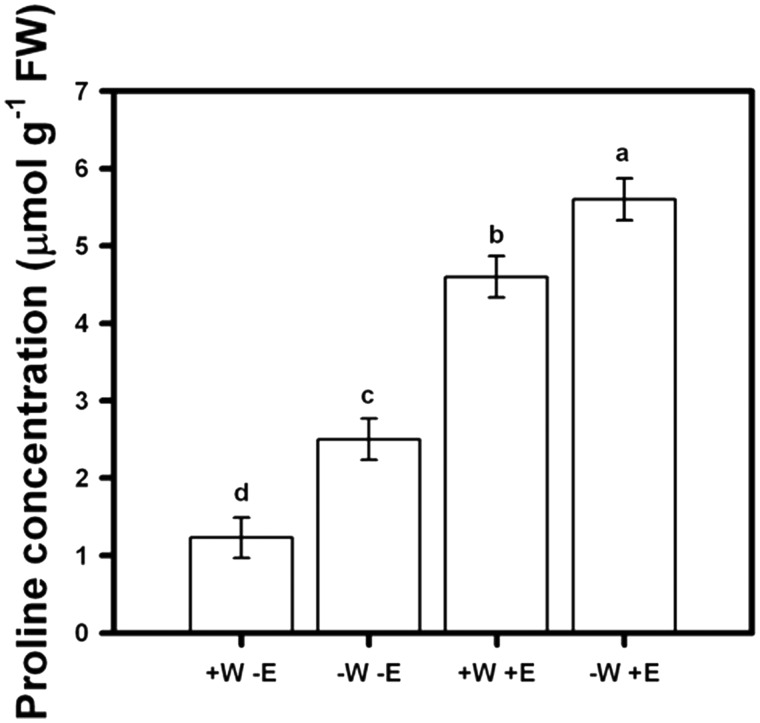
Proline concentration (mmol/g FW) of lettuce plants measured at 90 days and subjected to 40 and 30 ml/day of tap water (+W and −W), and with or without presence of root-endophytes (+E and −E) isolated from Antarctic plants. Different letters indicate significant differences; Tukey test, α < 0.05). Mean values are shown (± 1 SD).

Proline accumulation was significantly different among treatments (F_99, 3 _=_ _1264.12; *P* < 0.001), with the lowest concentration occurring in 100 % irrigation without root-endophytes (Fig. 2, Tukey test *P* < 0.05). A significant increase was recorded when plants were grown in 75 % irrigation without root-endophytes (Fig. 2, Tukey test *P* < 0.05). Conversely, when plants were inoculated with root-endophytes, an additional increase in their proline concentrations was registered in comparison to non inoculated plants (Fig. 2, Tukey test *P* < 0.05). Furthermore, the treatment with 75 % irrigation with addition of root-endophytes exhibited the highest accumulation of this osmolyte (Fig. 2, Tukey test *P* < 0.05). The water restriction regime (75 % irrigation) plus root-endophytes showed the highest proline accumulation compared to 100 % irrigation with or without endophytes. Endophyte inoculation by irrigation treatment interaction was statistically significant (Fig. 2, Tukey test *P* < 0.05).

**Figure 3:**
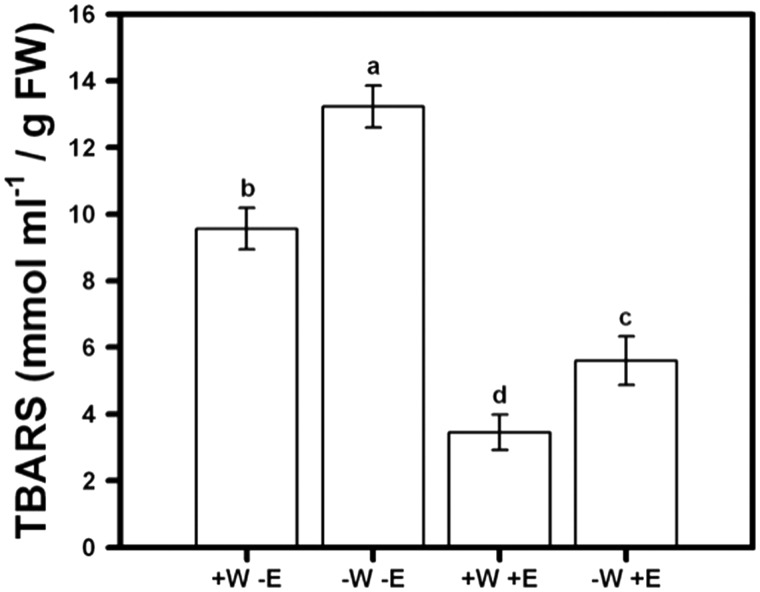
Peroxidation of lipids (TBARS) of lettuce plants measured at 90 days and subjected to 40 and 30 ml/day of tap water (+W and –W), and with or without presence of root-endophytes (+E and −E) isolated from Antarctic plants. Different letters indicate significant differences; Tukey test, α < 0.05). Mean values are shown (±1 SD).

Thiobarbituric acid reactive substances (TBARS) significantly differed between treatments with different water availability and root-fungal endophytes conditions (F_3, 99 _=_ _76.11; *P* = 0.024, Fig. 3). TBARS concentration of lettuce plants in symbiosis with endophytes was two times lower than when grown alone (Fig. 3, Tukey test *P* < 0.05). The interaction of water × endophyte was significant, because TBARS concentration increased in non-symbiosis treatments, but this increase was significantly greater under drought treatment (Fig. 3, Tukey test *P* < 0.05).

### Quantitative real-time PCR (qRT-PCR) analysis

**Figure 4:**
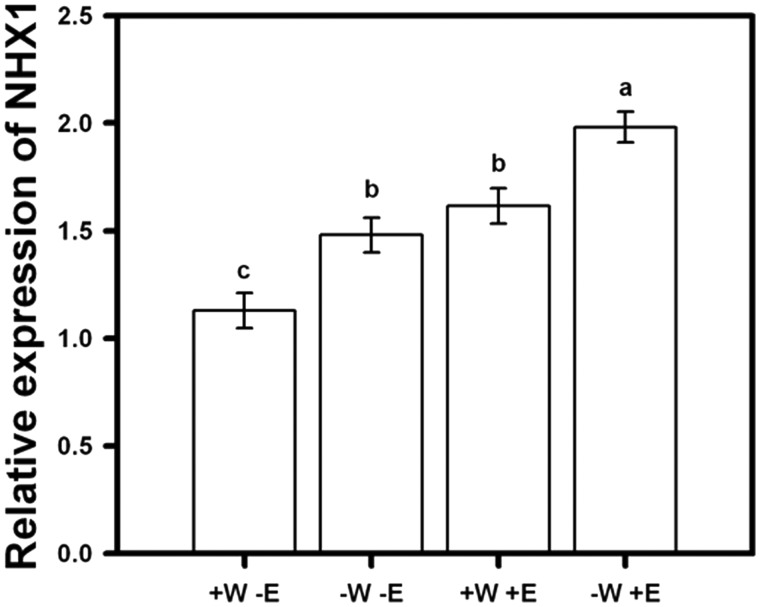
Expression levels of *NHX1* in shoot tissue of lettuce plants measured at 90 days and subjected to 40 and 30 ml/day of tap water (+W and −W), and with or without presence of root-endophytes (+E and −E) isolated from Antarctic plants. Different letters indicate significant differences; Tukey test, α< 0.05). Mean values are shown (±1 SD). The NHX1 is a gene belonging to family genes that code for vacuolar Na^+^/H^+^ antiporters, acting for vacuolar Na^+^ sequestration and pH regulation, both important for osmotic tolerance.

Transcript levels of *NHX1* in shoot tissues were significantly different among treatments (F_3, 99 _=_ _53.61; *P* = 0.039, Fig. 4), being highest in the 75 % irrigation plus root-endophytes treatment, followed by 100 % irrigation plus endophytes and 75 % irrigation without endophytes, and finally by 100 % irrigation without endophytes (Fig. 4). Overall, treatments with root-endophytes inoculation displayed significant changes in the regulation of *NHX1* under the experimental conditions in comparison to un-inoculated plants. By contrast, *NHX1* was similarly and significantly up-regulated by reduced irrigation in the treatment without endophytes compared with 100 % irrigation plus endophytes (Fig.4, Tukey test *P* < 0.05). The relative transcription of this gene was strongly induced by 75 % irrigation in root-endophytes inoculated plants (about 2-fold relative to 100 % irrigation with no endophytes) (Fig. 4, Tukey test *P* < 0.05).

### Nutrient content

Lettuce plants inoculated with Antarctic root-endophytes showed significantly greater percentage of nitrogen, potassium and calcium than non-inoculated plants (Table 2) in both 100 and 75 % irrigation. In contrast, magnesium was significantly increased in non-inoculated plants, while phosphorus not shown differences among treatments (Table 2). Nitrogen content was 1.4 times higher in those lettuce plants with presence of endophytes, and potassium and calcium content was 1.5 and 1.6 higher in plants associated to root-endophytes compared with non-inoculated plants (Table 2).

**Table 2.** Nutrient percentages in shoot tissues of lettuce plants on dry-weight basis.

Nutrient	+W −E	−W −E	+W +E	−W +E
Nitrogen (%)	3.5b (± 0.24)	3.0c (± 0.11)	4.5a (± 1.7)	4.4a (± 2.2)
Phosphorus (%)	0.5a (± 0.14)	0.4a (± 0.16)	0.4a (± 0.13)	0.5a (± 0.12)
Potassium (%)	6.0b (± 0.82)	5.3b (± 0.76)	8.6a (± 0.98)	9.1a (± 1.1)
Magnesium (%)	0.5a (± 0.03)	0.4b (± 0.04)	0.3b (± 0.02)	0.3b (± 0.03)
Calcium (%)	1.0b (± 0.13)	1.1b (± 0.16)	1.7a (± 0.08)	1.7a (± 0.13)

Individuals were subjected to 40 and 30 ml/day of tap water (+W and −W, respectively), and with or without presence of root-endophytes (+E and −E, respectively) isolated from antarctic plants. Different letters indicate significant differences; tukey test, α < 0.05). mean values are shown (± 1 SD).

## Discussion

We found that inoculation with root-endophytes isolated from Antarctic plants improved the ecophysiological performance and yield in lettuce, under normal and reduced irrigation. In addition, individuals subjected to water shortage decreased their ecophysiological performance, thus reducing their accumulation of fresh biomass. Nevertheless, presence of root-endophytes enabled the maintenance of high photosynthetic capacity and fresh biomass, even under water limitation, possibly due to the improved water use efficiency and osmoprotectant mechanisms registered in these individuals. Similar results have been found in other studies. For instance, Basahi *et al.* (2014) reported that the photosynthesis, transpiration rate and stomatal conductance in lettuce individuals were significantly reduced in response to reduction in water availability. Additionally, Karam *et al.* (2002) showed that different lettuce cultivars presented a lower number of leaves and a 39 % drop in fresh biomass, triggered by water deficit.

Overall, it has been shown that plants subject to water shortage allocate greater amount of their resources to roots until water uptake increases (Pessarakli, 2007; Kerbiriou *et al.* 2013). In our plants, although temporal variations in the root system as strategy to increase water uptake were not assessed, greater fresh root biomass and maintenance of the photosynthetic rate and high yield in water-limited plants could be attributed to the positive effect of endophytes at the root-level. It has been suggested that endophyte-induced variations in the rhizosphere, like production of sugars, proteins and/or enzymes that avoid cell damage in membranes could allow some crops to cope with water-limiting conditions (Baltruschat *et al.* 2008). Thus, endophyte inoculation in lettuce roots could be an efficient strategy to maintain high photosynthetic capacity as well as WUE and, hence, a high yield under water shortage and/or reduce water cost in lettuce crops. Similarly, it has been shown by previous studies (Jumpponen *et al.* 1998; Newsham, 2011; Mandyam and Jumpponen, 2015) as well as suggested by our findings, that symbiosis can facilitate nutrient uptake indicating that root-endophytes presence can be responsible for increases the N and P contents in tissue of inoculated plants.

In the four assessed treatments, proline did accumulate when seedlings were exposed to decreased water regime and inoculation with root-endophytes, irrespective of water regime in the latter case, suggesting that the presence of endophytes imposed a positive stimulus, even when plants were fully irrigated. Considering that this compatible solute acts as an osmoprotectant that mitigates abiotic stress (Szabados and Savouré, 2010) the proline induction by endophyte presence would be paramount to helping mitigate water restriction in lettuce plants. On the other hand, an alternative hypothesis could be considered to explain the concerted increase in proline contents and root system mediated by endophytes. The increase in proline could be a consequence of increased K content displayed by the inoculated plants. Proline accumulation could be a way to balance the cytoplasmic osmolarity in cells with large vacuoles filled with K. Furthermore, the enhanced K content could be a result of more robust and active root systems as those showed in inoculated plants. In addition, enhanced root system development could be a consequence of an increase in reduced carbon molecules generated by greater photosynthetic capacity displayed by the inoculated plants. Despite the evident positive effects of inoculation with endophytes on the photosynthetic rate and WUE in lettuce plants and expression of osmoprotectant compounds to cope with drought, more studies should be addressed to unravel the specific and concerted mechanisms by which Antarctic root-endophytes act.

Avoidance of cell damage by peroxidation of lipids could be considered a key mechanism to cope with drought and explain the greater fresh biomass and higher photosynthetic rate in lettuce plants inoculated with endophytes compared with non-inoculated plants subjected to drought. Our results are in agreement with previous studies since lettuce plants showed low TBARS production when in symbiosis with Antarctic root-endophytes in both well watered or drought condition. However, when lettuce plants were grown alone, the TBARS production significantly increased in all water conditions, suggesting that presence of root-endophytes is relevant to avoid cell damage. It has been suggested that cell damage induced by peroxidation of lipids can reduce growth (Tian and Lei, 2007) as was found in lettuce plants when grown in absence of root-endophytes, mainly when plants were subjected to drought. Thus, our results suggest that Antarctic root-endophytes induce great environmental tolerance in lettuce and help maintain fresh biomass accumulation by avoiding a decrease in physiological performance and fitness-related traits, likely modulated by cell damage.

Several functions have been associated with NHX antiporters, including the pH, Na^+ ^and K^+ ^homeostasis (Leidi *et al.* 2010; Bassil and Blumwald, 2014), cell expansion (Apse *et al.* 2003), salt tolerance (Hernandez *et al.* 2009; Bassil and Blumwald, 2014) and very recently microtubule organization and directional root growth (McCubbin *et al.* 2014). A generally accepted mode of NHX operation results in the transport of either K^+ ^or Na^+ ^into the vacuole or endosome in exchange for H^+ ^efflux to the cytosol (*NHX1*; Bassil and Blumwald, 2014), also contributing to K^+ ^uptake, capturing K^+ ^into vacuoles for cellular storage, turgor generation and pH regulation. Under salt or osmotic stress NHX proteins fulfil a protective function through the vacuolar compartmentalization of K^+ ^and, in some cases, of Na^+ ^thereby preventing toxic Na^+^–K^+ ^ratios in the cytosol while accruing solutes for osmotic balance. Hence, K^+ ^might posses a major osmotic role in plant cells, and vacuolar accumulation of this element is an especially crucial feature for plants under osmotic stress (Jiang *et al.* 2010). In our study, inoculated plants induced *NHX* transcripts, therefore stimulating K^+ ^accumulation and preventing cellular damage caused by osmotic stress.

### Final remarks

Water available for irrigation has been reduced (and could be more so) in most countries as a result of global climate change and the change in land use (IPCC, 2013). Therefore, knowledge related to the effect of root-endophytes on the physiological tolerance and productivity on lettuce crops could be a successful strategy to maximize water use efficiency and hence maintain an optimal yield in zones affected by desertification. Root-endophytes can improve plant performance by means of several different mechanisms. For instance, increases in abiotic and biotic tolerance have been proposed for other fungal root-endophytes such as *Geomyces* and *Lecanicillium* (Rosa *et al.* 2010). Nonetheless, more experimental evidence is needed to determinate the precise mechanisms by which *P. brevicompactum* and *P. chrysogenum* improve drought tolerance and ecophysiological performance in lettuce crops. Future studies should be conducted to unravel the possible mechanism(s) by which these fungal endophytes provide higher environmental tolerance, and to determine their potential as biotechnological tool for food security.

## Sources of Funding

This work was supported by grants from Fondo Nacional de Desarrollo Científico y Tecnológico de Chile (FONDECYT project number 11140607) and Iniciativa Científica Milenio (ICM project number NC120027). Isolation and identification of root-endophytes was partiality supported by FONDECYT project number 3140279.

## Contribution by the Authors

Marco A. Molina-Montenegro and Cristian Torres-Díaz conceived the idea. Marco A. Molina-Montenegro, Rómulo Oses, Andrés Zurita-Silva and Cristian Atala conducted the experiments. Marco A. Molina-Montenegro, Andrés Zurita-Silva and Simón Ruiz-Lara made valuable and significant comments on the manuscript.

## Conflict of Interest Statement

None declared.

## References

[plw062-B1] Acar B Paksoy M Türkmen Ö Seymen M. 2008 Irrigation and nitrogen level affect lettuce yield in greenhouse condition. African Journal of Biotechnology 24:4450–4453.

[plw062-B2] Apse MP Sottosanto JB Blumwald E. 2003 Vacuolar cation/H+ exchange, ion homeostasis, and leaf development are altered in a T-DNA insertional mutant of AtNHX1, the Arabidopsis vacuolar Na+/H+ antiporter. Plant Journal 36:229–239. 14535887 10.1046/j.1365-313x.2003.01871.x

[plw062-B3] Baltruschat H Fodor J Harrach BD Niemczyc E Barna B Gullner G Janeczko A Kogel KH Schäfer P Schwarczinger I Zuccaro A Skoczowski A. 2008 Salt tolerance of barley induced by the root endophyte *Piriformospora indica* is associated with a strong increase in antioxidants. New Phytologist 180:501–510. 18681935 10.1111/j.1469-8137.2008.02583.x

[plw062-B4] Basahi JM Ismail IM Hassan IA. 2014 Effects of enhanced UV-B radiation and drought stress on photosynthetic performance of Lettuce (*Lactuca sativa* L. Romaine) Plants. Annual Research and Review in Biology 4:1739–1756.

[plw062-B6] Bassil E Blumwald E. 2014 The ins and outs of intracellular ion homeostasis: NHX-type cation/H+ transporters. Current Opinion in Plant Biology 22:1–6. 25173972 10.1016/j.pbi.2014.08.002

[plw062-B7] Bates LS Walder RP Teare ID. 1973 Rapid determination of free proline for water-stress studies. Plant and Soil 39:205–207.

[plw062-B8] Boroujerdnia M Ansari NA Dehcordie FS. 2007 Effect of cultivars, harvesting time and level of nitrogen fertilizer on nitrate and nitrite content, yield in Romaine lettuce. Asian Journal of Plant Science 6:550–553.

[plw062-B9] Chang S Puryear J Cairney J. 1993 A simple and efficient method for isolating RNA from pine trees. Plant Molecular Biology Report 11:113–116.

[plw062-B10] Claeys H Inze D. 2013 The agony of choice: how plants balance growth and survival under water-limiting conditions. Plant Physiology 162:1768–1779. 23766368 10.1104/pp.113.220921 PMC3729759

[plw062-B11] Coleman-Derr D Tringe SG. 2014 Building the crops of tomorrow: advantages of symbiont-based approaches to improving abiotic stress tolerance. Frontiers in Microbiology 5:283. 24936202 10.3389/fmicb.2014.00283 PMC4047557

[plw062-B12] Collado J Platas G Pelaez F. 1996 Fungal endophytes in leaves, twigs and bark of *Quercus ilex* from Central Spain. Nova Hedwigia 63:347–360.

[plw062-B13] Drummond AJ Ashton B Buxton S, 2011 Geneious v5.4. http://www.geneious.com.

[plw062-B14] Egert M Tevini M. 2002 Influence of drought on some physiological parameters symptomatic for oxidative stress in leaves of chives (*Allium schoenoprasum*). Environmental and Experimental of Botany 48:43–49.

[plw062-B15] Estrada B Aroca R Maathuis FJM Barea JM Ruiz-Lozano JM. 2013 Arbuscular mycorrhizal fungi native from a Mediterranean saline area enhance maize tolerance to salinity through improved ion homeostasis. Plant, Cell and Environment 36:1771–1782. 10.1111/pce.12082 23421735

[plw062-B16] FAO. 2008 Land and Plant Nutrition Management Service. http://www.fao.org/ag/agl/agll/spush (22 December 2014).

[plw062-B17] Fardella C Oses R Torres-Díaz C Molina-Montenegro MA. 2014 Hongos endófitos antárticos como herramienta para la reintroducción de especies nativas en zonas áridas. Bosque 35:235–239.

[plw062-B18] Fedoroff NV Battisti DS Beachy RN, 2010 Radically rethinking agriculture for the 21st century. Science 327:833–834. 20150494 10.1126/science.1186834 PMC3137512

[plw062-B19] Flexas J Bota J Galmés J Medrano H Ribas-Carbó M. 2006 Keeping a positive carbon balance under adverse conditions: responses of photosynthesis and respiration to water stress. Physiologia Plantarum 127:343–352.

[plw062-B21] Grover M Ali SZ Sandhya V Rasul A Venkateswarlu B. 2011 Role of microorganism in adaptation of agriculture crops to abiotic stresses. World Journal of Microbiology and Biotechnology 27:1231–1240.

[plw062-B22] Hamdy A Ragab R Scarascia-Mugnozza E. 2003 Coping with water scarcity: water saving and increasing water productivity. Irrigation and Drainage 52:3–20.

[plw062-B23] Hernandez A Jiang X Cubero B, 2009 Mutants of the Arabidopsis thaliana cation/H+ antiporter AtNHX1 conferring increased salt tolerance in yeast: the endosome/prevacuolar compartment is a target for salt toxicity. Journal of Biological Chemistry 284:14276–14285. 19307188 10.1074/jbc.M806203200 PMC2682876

[plw062-B24] Hodges DM DeLong JM Forney CF Prange RK. 1999 Improving the thiobarbituric acid-reactive-substances assay for estimating lipid peroxidation in plant tissues containing anthocyanin and other interfering compounds. Planta 207:604–611. 10.1007/s00425-017-2699-3 28456836

[plw062-B25] IPCC. 2013 Intergovernmental Panel on Climate Change. http://www.ipcc.ch.

[plw062-B26] Jiang X Leidi E Pardo JM. 2010 How do vacuolar NHX exchangers function in plant salt tolerance? Plant Signal and Behaviour 8:792–795. 10.4161/psb.5.7.11767 PMC3014531 20495345

[plw062-B27] Jumpponen A Trappe JM Mattson KG Trappe JM. 1998 Mycorrhizal functioning of *Phialocephala fortinii*: interactions with soil nitrogen and organic matter. Mycorrhiza 7:261–265. 24578052 10.1007/s005720050190

[plw062-B28] Karam F Mounzer O Sarkis F Lahoud R. 2002 Yield and nitrogen recovery of lettuce under different irrigation regimes. Journal Applied Horticulture 4:70–76.

[plw062-B29] Kerbiriou PJ Stomph TJ Van Der Putten PEL Lammerts Van Bueren ET Struik PC. 2013 Shoot growth, root growth and resource capture under limiting water and N supply for two cultivars of lettuce (*Lactuca sativa* L.). Plant and Soil 371:281–297.

[plw062-B30] Lambers H Chapin FS Pons TL. 1998 Plant physiological ecology. Springer, Heidelberg 540 pp.

[plw062-B31] Leidi EO Barragan V Rubio L, 2010 The AtNHX1 exchanger mediates potassium compartmentation in vacuoles of transgenic tomato. Plant Journal 61:495–506. 19912566 10.1111/j.1365-313X.2009.04073.x

[plw062-B32] Livak KJ Schmittgen TD. 2001 Analysis of relative gene expression data using real-time quantitative PCR and the 2-ΔΔCT method. Methods 25:402–408. 11846609 10.1006/meth.2001.1262

[plw062-B33] Malinowski DP Belesky DP. 2000 Adaptations of endophyte-infected cool season grasses to environmental stresses: mechanisms of drought and mineral stress tolerance. Crop Science 40:923–940.

[plw062-B34] Mandyam KG Jumpponen A. 2015 Mutualism-parasitism paradigm synthesized from results of root-endophyte models. Frontiers in Microbiology 5:776. 25628615 10.3389/fmicb.2014.00776 PMC4290590

[plw062-B35] Martínez EA Veas E Jorquera C San Martín R Jara P. 2009 Re-introduction of *Chenopodium quinoa* Willd. into arid Chile: Cultivation of two lowland races under extremely low irrigation. Journal of Agronomy and Crop Science 195:1–10.

[plw062-B36] McCubbin T Bassil E Zhang S Blumwald E. 2014 Vacuolar Na+/H+ NHX-type antiporters are required for cellular K+ homeostasis, microtubule organization and directional root growth. Plants 3:409–426. 27135511 10.3390/plants3030409 PMC4844347

[plw062-B20] Molina-Montenegro MA Zurita-Silva A Oses R. 2011 Effects of water supply on physiological and yield production in Lettuce culture (Lactuca sativa L.). Ciencia e Investigación Agraria 12:23–29.

[plw062-B37] Newsham KK. 2011 A meta-analysis of plant responses to dark septate root endophytes. New Phytologist 190:783–793. 21244432 10.1111/j.1469-8137.2010.03611.x

[plw062-B38] Nissen J San Martín K. 2004 Uso de poliacrilamidas y el riego en el manejo hídrico de lechugas (*Lactuca sativa* L.). Agro Sur 32:1–12.

[plw062-B39] Nobel PS. 2005 Physicochemical and environmental plant physiology. Academic Press, New York, 582 pp.

[plw062-B40] Pessarakli M. 2007 Handbook of plant and crop physiology, 2nd ed Dekker Publisher, New York, 1000 pp.

[plw062-B41] Pfaffl MW. 2001 A new mathematical model for relative quantification in real-time RT-PCR. Nucleic Acids Research 29:2002–2007. 10.1093/nar/29.9.e45 PMC55695 11328886

[plw062-B42] Rosa LH Almeida-Vieira ML Furtado I Rosa CA. 2010 Endophytic fungal community associated with the dicotyledonous plant *Colobanthus quitensis* (Kunth) Bartl. (Caryophyllaceae) in Antarctica. FEMS Microbiology Ecology 73:178–189. 20455944 10.1111/j.1574-6941.2010.00872.x

[plw062-B43] Ruiz-Carrasco K Antognoni F Coulibaly AK, 2011 Variation in salinity tolerance of four lowland genotypes of quinoa (*Chenopodium quinoa* Willd.) as assessed by growth, physiological traits, and sodium transporter gene expression. Plant Physiology and Biochemistry 49:1333–1341. 22000057 10.1016/j.plaphy.2011.08.005

[plw062-B44] Ruiz-Lozano JM. 2003 Arbuscular mycorrhizal symbiosis and alleviation of osmotic stress. New perspectives for molecular studies. Mycorrhiza 13:309–317. 12690537 10.1007/s00572-003-0237-6

[plw062-B45] Ruiz-Lozano JM Porcel R Azcón S Aroca R. 2012 Regulation by arbuscular mycorrhizae of the integrated physiological response to salinity in plants: new challenges in physiological and molecular studies. Journal of Experimental Botany 63:4033–4044. 22553287 10.1093/jxb/ers126

[plw062-B46] Sánchez CHA. 2000 Response of lettuce to water and nitrogen on sand and the potential for leaching of nitrate-N. Horticulture Science 35:73–75.

[plw062-B47] Shabala L Mackay A Tian Y Jacobsen S-E Zhou D Shabala S. 2012 Oxidative stress protection and stomatal patterning as components of salinity tolerance mechanism in quinoa (*Chenopodium quinoa* Willd). Physiologia Plantarum 146:26–38. 22324972 10.1111/j.1399-3054.2012.01599.x

[plw062-B48] Sokal R Rohlf FJ. 1995 Biometry: the principles and practice of statistics in biological research. Freeman Company, New York, 887 pp.

[plw062-B49] Swarthout D Harper E Judd S Bultman T. 2009 Measures of leaf-level water-use efficiency in drought stressed endophyte infected and non-infected tall fescue grasses. Environmental and Experimental Botany 66:88–93.

[plw062-B50] Szabados L Savouré A. 2010 Proline: a multifunctional amino acid. Trends in Plant Science 15:89–97. 20036181 10.1016/j.tplants.2009.11.009

[plw062-B51] Thom ER Popay AJ Hume DE Fletcher LR. 2012 Evaluating the performance of endophytes in farm systems to improve farmer outcomes—a review. Crop and Pasture Science 63:927–943.

[plw062-B52] Tian XR Lei YB. 2007 Physiological responses of wheat seedlings to drought and UV-B radiation, effect of exogenous sodium nitroprusside application. Plant Physiology 54:676–682.

[plw062-B53] Upson R Read DJ Newsham KK. 2009 Nitrogen form influences the response of *Deschampsia antarctica* to dark septae root endophytes. Mycorrhiza 20:1–11. 19495811 10.1007/s00572-009-0260-3

[plw062-B54] Vilgalys R Hester M. 1990 Rapid genetic identification and mapping of enzymatically amplified ribosomal DNA from several Cryptococcus species. Journal of Bacteriology 172:4239–4246. 10.1128/jb.172.8.4238-4246.1990 PMC213247 2376561

[plw062-B55] White TJ Bruns T Lee S Taylor J. 1990 Amplification and direct sequencing of fungal ribosomal RNA genes for phylogenetics In: Innis MA Gelfand DH Sninsky JJ White TJ, eds. PCR protocols: a guide to methods and applications. New York: Academic Press, 315–322.

